# Mental Health of Transgender Youth: A Comparison of Assigned Female at Birth and Assigned Male at Birth Individuals

**DOI:** 10.3390/jcm12144710

**Published:** 2023-07-16

**Authors:** Diana Klinger, Stefan Riedl, Heidi Elisabeth Zesch, Sofia-Marie Oehlke, Sabine Völkl-Kernstock, Paul L. Plener, Andreas Karwautz, Oswald D. Kothgassner

**Affiliations:** 1 Department of Child and Adolescent Psychiatry, Medical University of Vienna, 1090 Vienna, Austria; 2 Comprehensive Center of Pediatrics, Medical University of Vienna, 1090 Vienna, Austria; 3 Department of Pediatrics and Adolescent Medicine, Medical University of Vienna, 1090 Vienna, Austria; 4 St. Anna Children’s Hospital, Medical University of Vienna, 1090 Vienna, Austria

**Keywords:** gender dysphoria, gender incongruence, transgender, mental health, adolescence

## Abstract

Gender dysphoric adolescents report a gender identity which is incongruent with their assigned sex at birth, whereby the experienced incongruence is accompanied by clinically relevant distress. The aim of the study was to assess and compare the mental health of transgender youth by assigned sex at birth. A total of n = 49 adolescents (n = 29 assigned females at birth, n = 20 assigned male at birth) aged 12 to 18 years with the diagnosis of gender dysphoria according to DSM-5 were included in the study. The adolescents underwent a psychological assessment in a child and adolescent psychiatry outpatient department prior to starting gender-affirming medical treatment, completing relevant mental health questionnaires. Although no differences were found in psychiatric disorders, more externalizing problems above the clinical threshold were reported by parents in assigned female at birth (AFAB) adolescents. On the other hand, internalizing problems, both in general and within the clinical range, were found to be more prevalent in assigned male at birth (AMAB) adolescents, as indicated by self-report. Our results suggest that a comprehensive assessment of mental health in gender dysphoric adolescents is crucial for understanding the diverse range of challenges they may face and tailoring appropriate interventions to address their specific needs.

## 1. Introduction

Transgender youth report a gender identity that does not correspond to their assigned sex at birth. In such cases, the experienced identity differs from the sexual characteristics, which leads to a gender incongruence (GI). If the experienced incongruence is associated with significant distress and functional impairment, one speaks of gender dysphoria (GD) [1]. In line with this, the terms GI/GD have also been adopted in the recent versions of the main diagnostic classification systems, the ICD-11 of the WHO and the DSM-5-TR of the American Psychiatric Association, replacing the former terms gender identity disorder and transsexualism, respectively [2,3].

According to the DSM-5-TR, GD in adolescents is characterized by a significant incongruence between one’s experienced/expressed gender and assigned gender that persists for at least 6 months. To meet the diagnosis, individuals must fulfill at least two of the six predefined criteria, including a strong desire to get rid of one’s primary and/or secondary sex characteristics, to be of the other gender, to be treated as the other gender, to possess the primary and/or secondary sexual characteristics of the other gender or to have a strong conviction of having the typical feelings and reactions of the other gender. Additionally, the symptoms are accompanied by clinically relevant suffering or impairment in social, educational or other important areas of functioning [2].

Gender dysphoric adolescents are significantly more likely to show psychopathological abnormalities than their cisgender peers, whose gender identity aligns with the sex they were assigned at birth. However, previous studies have reported substantial differences regarding the proportion of adolescents suffering from co-occurring mental health problems. Mustanski et al. [4] found that around one third of adolescents identifying themselves as transgender, individuals whose gender identity differs from the sex they were assigned at birth met the criteria for a co-occurring mental disorder. De Vries et al. [5] reported comparable rates; 32.4% of the adolescents with gender identity disorder had a concurrent psychiatric disorder. A more recent study also provided similar results; in the study by McCallion et al. [6], 37% of the gender dysphoric youth assessed had a mental health disorder. Meyenburg [7] reported a higher proportion of children and adolescents with psychopathological abnormalities who met the diagnostic criteria of a gender identity disorder according to DSM-IV. Of 268 children and adolescents that presented at the Frankfurt University Gender Identity Clinic from 1987 to 2013, 41% of the 120 male-to-female (MtF) patients and 44% of the 148 female-to-male (FtM) patients presented with significant psychopathological abnormalities. Furthermore, Kaltiala-Heino et al. [8] reported that 75% of adolescent applicants for legal and medical sex reassignment were in psychiatric treatment for a reason other than their GD, whereas two recent studies have found even higher rates (over 85%) of diagnosed mental health conditions in gender dysphoric adolescents [9,10].

Mental health problems have been found to be more prevalent in gender dysphoric youth not only as psychiatric diagnoses but more broadly in the form of suicidality and self-harm [8,11,12,13], with transgender males particularly affected [14].

Previous studies have shown that transgender youth also present more abnormalities than their peers if their mental health is measured by the severity of their emotional and behavioral problems, assessed with the Achenbach System of Empirically Based Assessment (ASEBA) [15]. The first comparative study regarding psychological functioning of transgender youth was carried out by Dutch and Canadian gender identity clinics, using the parent and self-report forms of the ASEBA, the Child Behavior Checklist (CBCL) and the Youth Self Report (YSR), respectively. The study showed that approximately 40% of adolescents from both The Netherlands and Canada had a significant impairment in their psychological functioning as measured by the YSR. Internalizing problems, characterized by inward-directed emotional and behavioral difficulties like anxiety and depression, were more common than externalizing problems, which encompass outwardly expressed behaviors such as aggression and rule-breaking. On average, emotional and behavioral problems in the clinical range were found to be more prevalent among Canadian youth than those from The Netherlands [16]. The study by de Graaf et al. [17] which included adolescents from four European specialist gender services (The Netherlands, Belgium, the UK and Switzerland) provided similar results and showed that adolescents from the UK had the highest prevalence of emotional and behavioral problems, followed by Switzerland and Belgium. The lowest levels of behavioral and emotional problems were observed among those from The Netherlands. A similar pattern was found across all four clinics regarding gender differences: assigned female at birth (AFAB) adolescents had more behavioral and externalizing problems that were in the clinical range according to the parent reports. Self-reports indicated that assigned male at birth (AMAB) adolescents had a higher prevalence of clinically significant internalizing problems. The findings of the study by Van Cauwenberg et al. [18] differ slightly from those of the two earlier studies. Although the percentage of adolescents who fell into the clinical range according to the parent report in terms of the internalizing, externalizing and total problem score was likewise higher than the percentage who were clinically salient according to the self-report, no significant difference was found when the internalizing, externalizing and total problem scores were compared between AFAB and AMAB adolescents equally whether considering the parent report or self-report.

Rates of self-harm and suicidality (ideation and behavior) in gender dysphoric adolescents has already been assessed in several studies using corresponding items of the CBCL and/or YSR [19,20,21,22]. Increased self-harm/suicidality was reported regarding gender-referred compared to cisgender adolescents in all studies, in most cases without significant differences in assigned sex at birth. De Graaf et al. [20] found that suicidality was consistently higher among AFAB youth.

In summary, considering the results of previous studies, it is evident that gender dysphoric adolescents represent a vulnerable group regarding their mental health. However, it should be noted that different inclusion criteria were used in these studies and that only certain psychopathological correlates were recorded, which then also led to large differences regarding results. This study aims to assess the mental health, including emotional and behavioral problems of transgender youth undergoing psychological assessment at an Austrian child and adolescent psychiatry, with the primary objective of gaining a deeper understanding of the psychopathological correlates in transgender youth, specifically examining differences between AFAB and AMAB individuals. This is of significant importance as the number of adolescents seeking medical treatment for GD is increasing exponentially [23]. In this context, mental health outcomes are particularly important to inform medical treatment, and the recommendations of the new version of the Standards of Care indicate that mental problems that may interfere with diagnostic clarity, capacity to consent and/or gender-affirming medical treatments should be addressed [24].

## 2. Materials and Methods

### 2.1. Participants and Procedure

Participants were adolescents (12–18 years old) who presented to the outpatient department at the Department of Child and Adolescent Psychiatry of the Medical University of Vienna, Austria, for a psychological assessment before the initiation of a gender-affirming medical treatment (i.e., puberty suppression and/or gender-affirming hormone treatment) between October 2018 and January 2023. Diagnostic and treatment procedures followed the Austrian recommendations for the treatment process for gender dysphoria in children and adolescents. According to the recommendations, children and adolescents should complete an assessment by a child and adolescent psychiatrist, clinical psychologist, psychotherapist and pediatric endocrinologist for confirmation of a diagnosis of gender dysphoria in order to receive medical treatment. The psychological assessment should include a specific assessment of GD, differential diagnostics of related conditions and assessment of co-occurring disorders [25].

The study procedure also conformed to the psychological assessment in routine care for gender dysphoria at the clinic. At the initial evaluation including 4 to 5 appointments, demographic data, gender identity development and history of GD were assessed with a semi-structured interview including an anamnesis carried out with the adolescent and their legal guardian as well as with the adolescent alone. The participants and their legal guardians were also asked to fill out different questionnaires regarding mental health, psychosocial functioning, gender identity and body image. Cognitive functioning was measured using the German version of the Wechsler Intelligence Scale for Children [26]. A specific evaluation of executive functioning was performed only in case of suspected attention deficit hyperactivity disorder. All assessment visits were conducted by a trained clinical psychologist.

Inclusion criteria for the study were (1) an age between 12 and 18 years; (2) fulfilling the diagnostic criteria for GD according to DSM-5 at the time of the assessment. Exclusion criteria were defined as presence of a diagnosis of a (1) severe psychiatric disorder, such as schizophrenia, schizotypal or delusional disorder, and (2) difference of sex development. Participation was voluntary and was not rewarded, and written informed consent was obtained both from the participants and from their legal guardians. The study received approval from the ethics committee of the Medical University of Vienna (no. 1618/2017).

### 2.2. Measures

#### 2.2.1. Demographics and Clinical Characteristics

From the collected sociodemographic information, the following variables were included in the analysis: age at baseline, sex assigned at birth and self-reported gender identity. The latter was assessed with the open-ended question: “How would you identify yourself regarding your gender identity?” The marital status of the parents and living situation of the adolescents were divided into two categories: “living with both parents” (married, in cohabitation) vs. “other” (divorced, separated, parent deceased, child welfare foster placement/adopted).

#### 2.2.2. Gender Dysphoria and Co-Occurring Psychiatric Disorders

Fulfillment of the diagnostic criteria for GD was recorded by the clinical psychologist using a checklist. By employing the checklist, the presence of each diagnostic criterion was assessed based on the medical history, the semi-structured interview with the adolescent and the completed questionnaires. Previous psychiatric history and medical reports were recorded during the anamnesis interview and filed in the patient’s medical record. Diagnoses were assigned by the clinical psychologist according to ICD-10 at the end of the assessment process.

#### 2.2.3. Psychological Functioning

In order to assess the emotional and behavior problems, the German version of the Achenbach System of Empirically Based Assessment were used [15]. The Child Behavior Checklist (CBCL/6–18R) consists of 113 items and is used for external assessment by parents or legal guardians, whereas the Youth Self Report (YSR/11–18R) is a self-report questionnaire, which consists of 112 items [27]. All items are rated on a 3-point Likert-scale ranging from 0 = “not true” to 2 = “very true or often true”. Higher scores indicate greater severity regarding the corresponding problems. Psychological functioning was assessed through the eight syndrome scales, (1) Anxious/depressed; (2) Withdrawn/depressed; (3) Somatic complaints; (4) Social problems; (5) Thought problems; (6) Attention problems; (7) Rule-breaking behavior and (8) Aggressive behavior, as well as through the three higher order scales: Internalizing problems, Externalizing problems and the Total problem scale.

In this study, the following variables from the CBCL and YSR were used: the T-scores for (1) the eight syndrome scales; (2) Total problem score; (3) Internalizing problems; (4) Externalizing problems and (5) the clinical range scores for these scales. T-scores from the population-based, age- and gender-specific German norms were used. For both the CBCL and YSR, a Suicidality Index was calculated based on two items: “Deliberately harms self or attempts suicide” (Item 18) and “Talks about killing self” (Item 91). First, a sum score of the two items was computed; second, both items were dichotomized as either present (rated 1 or 2) or absent (rated 0) [19].

### 2.3. Data Analysis

All analyses were conducted in IBM SPSS Statistics version 28 (IBM Corporation, Armonk, NY, USA, 2021). Descriptive statistics are reported for all variables. Means and standard deviations (SDs) were used to summarize continuous variables, frequencies and percentages to summarize categorical variables. Demographics, clinical characteristics of AFAB and AMAB individuals were compared using independent samples *t*-tests for continuous variables and chi-square statistics/Fisher’s exact test for categorical variables. *p* < 0.05 was considered to indicate a statistically significant difference.

## 3. Results

### 3.1. Demographics and Clinical Characteristics

Table 1 provides an overview of the demographic and clinical characteristics of the sample. Forty-nine participants were enrolled in the study, and twenty-nine (59.2%) were assigned female at birth (AFAB), all identifying themselves as “male” or “transmale”. Twenty (40.8%) were assigned male at birth (AMAB), all individuals with a “female” or “transfemale” gender identity. Mean participant age was 15.02 years (range 12–17, SD = 1.32) at the first assessment. AFAB adolescents averaged 14.79 years of age (range 12–17, SD = 1.08), whereas AMAB adolescents averaged 15.35 years of age (range 12–17, SD = 1.57). All participants met the diagnostic criteria for gender dysphoria according to DSM-5. About half of the adolescents were living with both parents (51.0%), while the other half lived in other circumstances (49.0%). There was no significant difference between AFAB and AMAB youth in this regard.

### 3.2. Psychiatric Diagnoses

Of the 49 adolescents, 28 (57.1%) received at least one psychiatric diagnosis at the end of the assessment process apart from GD; 30.6% (n = 15) of adolescents were diagnosed with one, 24.5% (n = 12) with two and 2% (n = 1) with three co-occurring mental health disorders. There was no statistically significant difference between AFAB and AMAB adolescents in the number of diagnoses.

Table 2 summarizes the assigned diagnoses by categories. The majority had a diagnosis in the ICD-10 F4x diagnostic category (30.6%), mainly social phobia, followed by F3x (24.5%), primarily depressive disorders. Diagnoses from the category F9x (12.2%) were also present, including one AFAB adolescent diagnosed with ADHD and another with hyperkinetic conduct disorder. Furthermore, two AFAB youth and one AMAB youth had a tic disorder. All diagnoses from the F8x category (10.2%) were classified as Asperger syndrome. There was also no significant difference between gender dysphoric adolescents by sex assigned at birth regarding the diagnostic categories.

### 3.3. Psychological Functioning

Regarding the CBCL syndrome scales, there were significant differences in the T-scores in the rule-breaking and aggressive behavior scales between gender dysphoric AFAB and AMAB youth. AFAB adolescents showed significantly more rule-breaking and aggressive behavior according to parent reports than AMAB adolescents. T-scores in the scales Anxious/depressed, Withdrawn/depressed, Somatic complaints, Social problems, Thought problems and Attention problems, as well as in the higher order scales (Internalizing, Externalizing and Total problems) did not significantly differ between groups in CBCL. In the self-report, a significant difference was found in the higher order scale Internalizing problems: AMAB youth experienced more internalizing problems than their AFAB peers. Mean differences in the T-scores of the eight syndrome scales, the Externalizing and Total problems scales of the YSR were not statistically significant. Detailed results of the t-tests are reported in Table 3.

The results regarding the proportion of adolescents scoring in the clinical range support the findings described above. Fisher’s exact test showed that there was a significant association between the assigned sex assigned at birth and the percentage of adolescents with scores in the clinical range in the CBCL Externalizing problem scale (*p* = 0.015) with significantly more AFAB than AMAB youth within the clinical range. Furthermore, a chi-square test of independence revealed a significant relationship between sex assigned at birth and the percentage of adolescents scoring in the clinical range regarding the Internalizing problem scale of the YSR, χ^2^(1) = 7.21, *p* = 0.007. AMAB adolescents were more likely to score in the clinical range than AFAB adolescents. Neither concerning the T-scores in the CBCL nor in the YSR was there any further significant association between the sex assigned at birth and the proportion of adolescents in the clinically abnormal range in the eight syndrome scales and in the remaining higher order scales.

As for the calculated Suicidality Index, there were no significant differences regarding assigned sex at birth in either the CBCL or the YSR. After dichotomization of the Items 18 “Deliberately harms self or attempts suicide” and 91 “Talks about killing self” a significant association between a positive response to Item 18 in the YSR and sex assigned at birth was found, χ^2^(1) = 4.40, *p* = 0.036. AFAB individuals were more likely to respond positively to the item than AMAB individuals.

## 4. Discussion

The purpose of this study was to assess the mental health, including emotional and behavioral problems, of transgender youth in Austria, with a specific focus on examining differences between AFAB and AMAB individuals. Our sample consisted of 49 transgender adolescents, 29 transmale and 20 transfemale, who met the diagnostic criteria for gender dysphoria according to DSM-5.

Consistent with previous research, we found that more than half of the adolescents in our sample were diagnosed with co-occurring mental health disorders, with most of them receiving one or two diagnoses. Interestingly, our study recorded significantly higher rates of co-occurring mental health disorders compared to Mustanski et al. [4] and somewhat higher rates compared to Meyenburg et al. [7] and Spack et al. [28]. Similar to previous studies, we found that internalizing disorders, such as depressive and anxiety disorders, were the most common among adolescents with GD [12,13,29,30]. The most frequent diagnoses in our study were from the ICD-10 F4x diagnostic category, predominantly social phobia, followed by the F3x category, mostly depressive disorders.

When considering the development of internalizing disorders in transgender youth, several factors are frequently discussed. A widely recognized hypothesis posits that gender minority stress plays a significant role in the development of internalizing disorders, as transgender individuals often face significant stressors related to their gender identity, including societal stigma, discrimination and rejection [30,31,32]. The experience of GD itself can contribute to distress; moreover, invalidation and lack of acceptance by family, peers and society can lead to chronic stress, which increases the risk of internalizing disorders [30,33]. Another key aspect may also be that adolescence is a time for identity exploration and disclosure. The process of questioning, exploring and disclosing one’s gender identity can be emotionally challenging. Difficulties in navigating these processes can lead to increased vulnerability to internalizing disorders [34]. The study by Furente et al. [35] provides an additional explanation, showing that adolescents with GD demonstrate higher rates of social introversion (SI) compared to cisgender adolescents, despite similar levels of depressive and internalizing symptoms. Moreover, SI was identified as a predictor of internalizing symptomatology in AFAB adolescents with GD but not in cisgender adolescents. These findings highlight the need for further investigation into the link between GD and SI.

Our findings regarding the presence of autism spectrum disorders (ASD) in the sample, with 10.2% having an Asperger syndrome, are consistent with previous research indicating a higher prevalence of ASD in transgender youth compared to cisgender youth [30,36,37,38].

We found no difference in psychiatric diagnoses between AFAB and AMAB adolescents, both in terms of quantity and diagnostic categories. Our results are consistent with those reported in previous studies in this regard [8,29].

With regard to the psychological functioning, a difference between AFAB and AMAB youth was found. Parents reported more rule-breaking and aggressive behavior in AFAB adolescents than in AMAB adolescents in CBCL. We also observed a tendency towards statistical significance for AFAB youth concerning attention problems in the corresponding subscale of the CBCL. Complementing the parent report, AMAB adolescents stated in the YSR more internalizing problems than did AFAB youth. The results regarding the percentage of adolescents who scored in the clinical range correspond to the results already mentioned. There were significantly more AFAB than AMAB youth within the clinical range in the CBCL Externalizing problem scale; on the other hand, AMAB adolescents were more likely to score in the clinical range in the Internalizing problems scale of the YSR than AFAB adolescents. These results correlate well with the findings of de Graaf et al. [17] and may be interpreted as a reversal of the relationship between internalizing versus externalizing problems, in line with the gender-typical pattern of more internalizing problems in assigned female and more externalizing problems in assigned male cisgender youth [16]. We also found a significant difference concerning suicidality and self-injurious behavior: AFAB individuals were more likely to affirm Item 18 (“Deliberately harms self or attempts suicide”) in the YSR than AMAB individuals, which is in agreement with the findings of Holt et al. [13] and contradicts the findings of Edwards-Leeper et al. [21]. According to the review of Wittlin et al. [30], research has shown that transmasculine youth have higher rates of suicidality and self-harm compared to transfeminine youth. The underlying causes of these disparities have not been thoroughly examined, but in specific samples, transmasculine youth have reported diminished familial connection and heightened feelings of insecurity at home. Additionally, they have encountered increased discrimination, lack of affirmation, higher rates of nondisclosure and, in certain instances, lower levels of pride in their identity. Furthermore, AFAB adolescents demonstrate greater similarity to their cisgender peers in terms of nonsuicidal self-injury (NSSI) and suicidality. This finding suggests the potential significance of biological factors in relation to NSSI and suicidality [39]. Moreover, it emphasizes the need for targeted interventions, mental health support and suicide prevention strategies that specifically cater to the unique experiences and challenges faced by AFAB adolescents with GD.

This study has several strengths, one of which is the comprehensive assessment of mental health, which included multiple methods such as parent reports and self-reports. Additionally, diagnoses were assigned and recorded as part of the study rather than being obtained retrospectively or from different sources. Moreover, this is the first study in Austria to examine the mental health of transgender youth in general. Prior research has predominantly focused on identity development and the effectiveness of suicide prevention programs within this population. Therefore, this study fills an important gap in the literature by providing valuable insights into the overall mental health of transgender youth in Austria [40,41,42]. However, there are some limitations to consider, including the small sample size. Nevertheless, it is worth noting that the study includes the latest data available from Austria, which enhances the relevance and currency of our findings. Another limitation is that the sample does not include any non-binary transgender youth; thus, we were unable to gather information about this specific group.

Our results indicate that when assessing children with gender dysphoria, it would be reasonable for mental health professionals to routinely assess the presence of behavioral and emotional problems, as well as evaluate whether suicidality and self-harm are present. An accurate and comprehensive psychological assessment is crucial as it ensures that any mental health concerns the adolescent may have, which could potentially affect diagnostic clarity, capacity to consent and/or gender-affirming medical treatments, are addressed prior to the initiation of treatment. When considering adolescents with GD, particularly those who are neurodivergent, it becomes apparent that offering additional support, structured assistance and psychoeducation and allowing ample time is crucial before making decisions concerning gender-affirming medical treatments [24]. Regarding suicidality and self-harm, more sensitive measurements with multiple items and a wider range of severity, such as the Functional Assessment of Self-Mutilation [43] or the Self-Injurious Thoughts and Behavior Interview–Revised [44], would be recommended. This is a particularly relevant issue as suicidality may interfere with capacity for consent. Providing appropriate care should be prioritized to adequately address threats or crises and allow the necessary time and stability for assessment and decision-making regarding treatment.

Future studies are necessary to advance our understanding of mental health disorders in gender dysphoric adolescents, with a particular focus on elucidating the differences between AFAB and AMAB individuals. It is crucial to investigate which mental disorders are reactive to or independent of GI/GD, considering factors such as perceived minority stress or underlying biological determinants. Additionally, research should explore which mental disorders are more likely to remit after affirmative treatment, providing valuable insights into effective intervention strategies. Gaining this nuanced insight will contribute to the development of comprehensive and tailored mental health care approaches for gender dysphoric adolescents.

## Figures and Tables

**Table 1:** Demographics and clinical characteristics.

	AFAB Adolescents n = 29 (59.2%)		AMAB Adolescents n = 20 (40.8%)		Total n = 49 (100%)	
	*M*	SD	*M*	SD	*M*	SD
**Age at first assessment**	14.79	1.08	15.35	1.57	15.02	1.32
	n	%	n	%	n	%
**Parental marital status and living situation**						
Living with both parents	16	32.7	9	18.4	25	51.0
Other	13	18.4	11	22.4	24	49.0

**Table 2:** Psychiatric diagnoses.

Diagnoses according to ICD-10 (n (%))	AFAB Adolescents n = 29 (59.2%)	AMAB Adolescentsn = 20 (40.8%)	Total n = 49 (100%)
F3x Mood (affective) disorders	6 (12.2%)	6 (12.2%)	12 (24.5%)
F4x Neurotic, stress-related and somatoform disorders	8 (16.3%)	7 (14.2%)	15 (30.6%)
F5x Behavioral syndromes associated with physiological disturbances and physical factors	-	1 (2%)	1 (2%)
F6x Disorders of adult personality and behavior	1 (2%)	-	1 (2%)
F8x Disorders of psychological development	1 (2%)	4 (8.2%)	5 (10.2%)
F9x Behavioral and emotional disorders with onset usually occurring in childhood and adolescence	4 (8.2%)	2 (4%)	6 (12.2%)

**Table 3:** Ratings on psychological functioning on the CBCL and YSR.

	**AFAB Adolescents**		**AMAB Adolescents**		***t (*** **47)**	***p***
	M *	**SD**	M *	**SD**		
**CBCL Scales**						
Anxious/depressed	64.07	12.72	63.40	11.17	0.190	0.850
Withdrawn/depressed	66.21	14.48	67.60	10.07	−0.372	0.712
Somatic complaints	60.03	11.03	61.35	9.01	−0.441	0.661
Social problems	58.07	8.53	57.75	8.20	0.131	0.897
Thought problems	62.90	8.94	61.30	6.99	0.669	0.507
Attention problems	60.79	9.17	56.75	6.24	1.836	0.073
Rule-breaking behavior	59.28	8.27	53.70	5.38	2.859	0.006
Aggressive behavior	58.48	10.46	53.30	3.92	2.433	0.020
Internalizing problems	62.93	14.19	67.70	10.93	−1.265	0.212
Externalizing problems	55.97	12.36	50.65	7.84	1.700	0.096
Total problems	62.86	12.03	60.65	7.82	0.722	0.474
**YSR Scales**						
Anxious/depressed	62.79	12.15	69.00	11.42	−1.800	0.078
Withdrawn/depressed	63.90	13.48	68.35	12.23	−1.179	0.244
Somatic complaints	58.31	9.63	62.35	7.45	−1.577	0.121
Social problems	59.10	9.06	62.30	7.82	−1.282	0.206
Thought problems	63.86	11.98	66.80	9.93	−0.903	0.371
Attention problems	61.55	11.04	63.05	10.49	−0.476	0.636
Rule-breaking behavior	57.07	8.79	56.15	8.65	0.362	0.719
Aggressive behavior	54.31	5.64	53.40	4.17	0.614	0.542
Internalizing problems	60.66	13.65	68.85	9.75	−2.306	0.026
Externalizing problems	53.55	9.78	54.05	7.56	−0.0192	0.849
Total problems	60.55	11.64	64.10	9.13	−1.141	0.260

* Mean values of the T-scores from the population-based, age- and gender-specific German norms.

## Data Availability

The datasets generated and analyzed during the current study are available from the corresponding author on reasonable request.
